# Cysteine Cathepsins in Breast Cancer: Promising Targets for Fluorescence-Guided Surgery

**DOI:** 10.1007/s11307-022-01768-4

**Published:** 2022-08-24

**Authors:** Daan G. J. Linders, Okker D. Bijlstra, Laura C. Fallert, Denise E. Hilling, Ethan Walker, Brian Straight, Taryn L. March, A. Rob P. M. Valentijn, Martin Pool, Jacobus Burggraaf, James P. Basilion, Alexander L. Vahrmeijer, Peter J. K. Kuppen

**Affiliations:** 1 Department of Surgery, Leiden University Medical Center, 2333 ZA Leiden, The Netherlands; 2 Department of Biomedical Engineering, Case School of Engineering, Case Western Reserve University, Cleveland, OH 44106 USA; 3 Akrotome Imaging, Charlotte, NC USA; 4 Department of Clinical Pharmacy and Toxicology, Leiden University Medical Center, 2333 ZA Leiden, The Netherlands; 5 Centre for Human Drug Research, 2333 CL Leiden, The Netherlands; 6 Leiden Academic Center for Drug Research, 2333 AL Leiden, The Netherlands; 7 Department of Radiology, Case School of Medicine, Case Western Reserve University, Cleveland, OH 44106 USA

**Keywords:** Cysteine cathepsins, Breast cancer, Targeted molecular imaging, Fluorescence-guided surgery, Near-infrared fluorescence imaging

## Abstract

The majority of breast cancer patients is treated with breast-conserving surgery (BCS) combined with adjuvant radiation therapy. Up to 40% of patients has a tumor-positive resection margin after BCS, which necessitates re-resection or additional boost radiation. Cathepsin-targeted near-infrared fluorescence imaging during BCS could be used to detect residual cancer in the surgical cavity and guide additional resection, thereby preventing tumor-positive resection margins and associated mutilating treatments. The cysteine cathepsins are a family of proteases that play a major role in normal cellular physiology and neoplastic transformation. In breast cancer, the increased enzymatic activity and aberrant localization of many of the cysteine cathepsins drive tumor progression, proliferation, invasion, and metastasis. The upregulation of cysteine cathepsins in breast cancer cells indicates their potential as a target for intraoperative fluorescence imaging. This review provides a summary of the current knowledge on the role and expression of the most important cysteine cathepsins in breast cancer to better understand their potential as a target for fluorescence-guided surgery (FGS). In addition, it gives an overview of the cathepsin-targeted fluorescent probes that have been investigated preclinically and in breast cancer patients. The current review underscores that cysteine cathepsins are highly suitable molecular targets for FGS because of favorable expression and activity patterns in virtually all breast cancer subtypes. This is confirmed by cathepsin-targeted fluorescent probes that have been shown to facilitate *in vivo* breast cancer visualization and tumor resection in mouse models and breast cancer patients. These findings indicate that cathepsin-targeted FGS has potential to improve treatment outcomes in breast cancer patients.

## Introduction

To date, breast cancer remains the most frequently diagnosed cancer and the leading cause of cancer-related mortality in women worldwide, representing about 25% of all cancer cases and 15% of all cancer deaths [1]. Most newly diagnosed breast cancer patients can be treated with breast-conserving surgery (BCS) combined with adjuvant radiation therapy [2–5]. Although such treatment offers better cosmetic results and equivalent survival outcomes compared with total mastectomy, BCS is associated with an increased risk of tumor-positive resection margins [5–9]. Margin status in turn is a critical determinant of local recurrence and in some cases disease-specific mortality [7–11].

BCS based on visual and tactile feedback assisted by current localization techniques, such as implanted radioactive iodine seeds, still results in tumor-positive resection margin rates up to 40% [12–15]. A tumor-positive resection margin after BCS necessitates re-resection or boost radiation therapy, resulting in worse cosmetic outcomes and increased morbidity, complication risks, and healthcare costs [16–18]. Evidently, there is an unmet need for a method to detect tumor-positive margins at the time of surgery to guide immediate resection of residual tumor tissue and prevent additional mutilating treatments.

Numerous pathology and imaging methods for intraoperative guidance and margin assessment have been evaluated to decrease tumor-positive resection margin rates after BCS, but most have significant clinical and technical limitations that have precluded widespread adoption [15, 19]. An ideal method for margin assessment during BCS would be able to detect tumor-positive margins rapidly, non-invasively, in real-time with high spatial accuracy. A technique that could meet these requirements is intraoperative tumor-targeted near-infrared (NIR) fluorescence imaging (FI).

Tumor-targeted NIR fluorescence-guided surgery combines the administration of a contrast agent, consisting of a fluorophore and a targeting moiety, with the use of a fluorescence-sensitive camera, matched to operate in the range of NIR fluorescence light (700–900 nm) [20]. It allows for rapid, real-time optical imaging of large surface areas by selectively highlighting cells that overexpress certain molecular targets.

In BCS, tumor-targeted NIR FI for tumor-positive resection margin detection could be used both *in vivo* on the resection cavity surfaces and *ex vivo* on the resected specimen [21–23]. However, due to the soft, pliable nature of a resected breast cancer specimen, its geometry does no longer accurately correspond to that of the resection cavity [24]. Consequently, correlating an *ex vivo* detected tumor-positive margin to the *in vivo* location of the residual tumor that needs to be excised is extremely difficult. Therefore, detection of residual tumor tissue on the surgical cavity walls seems to be the most promising approach.

Multiple contrast agents directed at different molecular targets are under extensive investigation for NIR fluorescence-guided BCS [25]. However, the cathepsin-targeted contrast agents are the most developed subgroup that has been shown to enable *in vivo* margin assessment in breast cancer patients [21, 23, 26–29].

Cathepsins are an important group of proteolytic enzymes that play a major role in both normal cellular physiology and disease [30, 31]. They are categorized according to the catalytic amino acids in their active site as either serine (cathepsin A and G), aspartic (cathepsin D and E), or cysteine (cathepsin B, C, F, H, K, L, O S, V, W, and X) proteases. This review focuses on the largest and best-studied group in breast cancer, the cysteine cathepsins. The cysteine cathepsin family in humans comprises 11 members that are involved in numerous intra- and extracellular processes [32]. These proteases are synthesized as pro-enzymes that are activated under mildly acidic conditions. However, not all members are equally dependent on pH and they differ in cellular location, tissue expression, and substrate specificity [30, 33, 34].

Cysteine cathepsins are mainly, but not exclusively, located within the endo-lysosomal system, where they are crucial for lipid and protein metabolism, autophagy, and antigen presentation [35, 36]. In addition, extra-lysosomal cathepsins located in the nucleus and mitochondrial matrix contribute to cell-cycle control and apoptosis initiation [33, 37]. Secreted cysteine cathepsins have been shown to participate in extracellular matrix (ECM) remodeling by degrading abundant components such as collagen and fibrin [34]. Most cysteine cathepsins like B, C, F, H, and L are expressed ubiquitously and share a broad spectrum of substrates [33, 34]. In contrast, cathepsin K and S are rather substrate specific and are expressed by certain cell types only [38–40]. The key role cysteine cathepsins play in this broad range of biochemical processes indicates that these proteases are essential for normal tissue homeostasis.

Dysregulated activity of cysteine cathepsins is associated with various pathological conditions, including atherosclerosis, neurodegenerative disease, osteoporosis, arthritis, and cancer [41–46]. In numerous cancer types, increased cysteine cathepsin enzymatic activity drives tumor progression, proliferation, invasion, and metastasis through a variety of different mechanisms [47, 48]. Given their crucial contribution to protein catabolism, it is plausible that cancer cells utilize cathepsins to meet their increased metabolic need [30, 49–52]. Additionally, secreted cathepsins mediate ECM degradation to facilitate cancer invasion and dissemination [47, 49]. There is also increasing evidence that the proteolytic products of extracellular molecules targeted by cathepsins, such as receptors and cell adhesion molecules, can induce cancer promoting signaling cascades [49].

During neoplastic transformation, the normally tightly regulated activity of cysteine cathepsins is altered by gene amplifications and the formation of transcript variants [53]. Another interesting phenomenon frequently reported is a shift in ratio between cysteine cathepsins and their endogenous inhibitors, such as cystatins and stefins, resulting in cathepsin upregulation [54]. This aberrant cathepsin activity is not restricted to one particular cancer type and occurs in both tumor cells as well as tumor-associated cells such as fibroblasts, myoepithelial cells, endothelial cells, and various immune cells, particularly tumor-associated macrophages (TAMs) [53]. Their expression pattern in tumor tissue and the extent to which they are upregulated varies between different types of cancer, stressing the importance of research specific to cancer type [55].

**Fig. 1:**
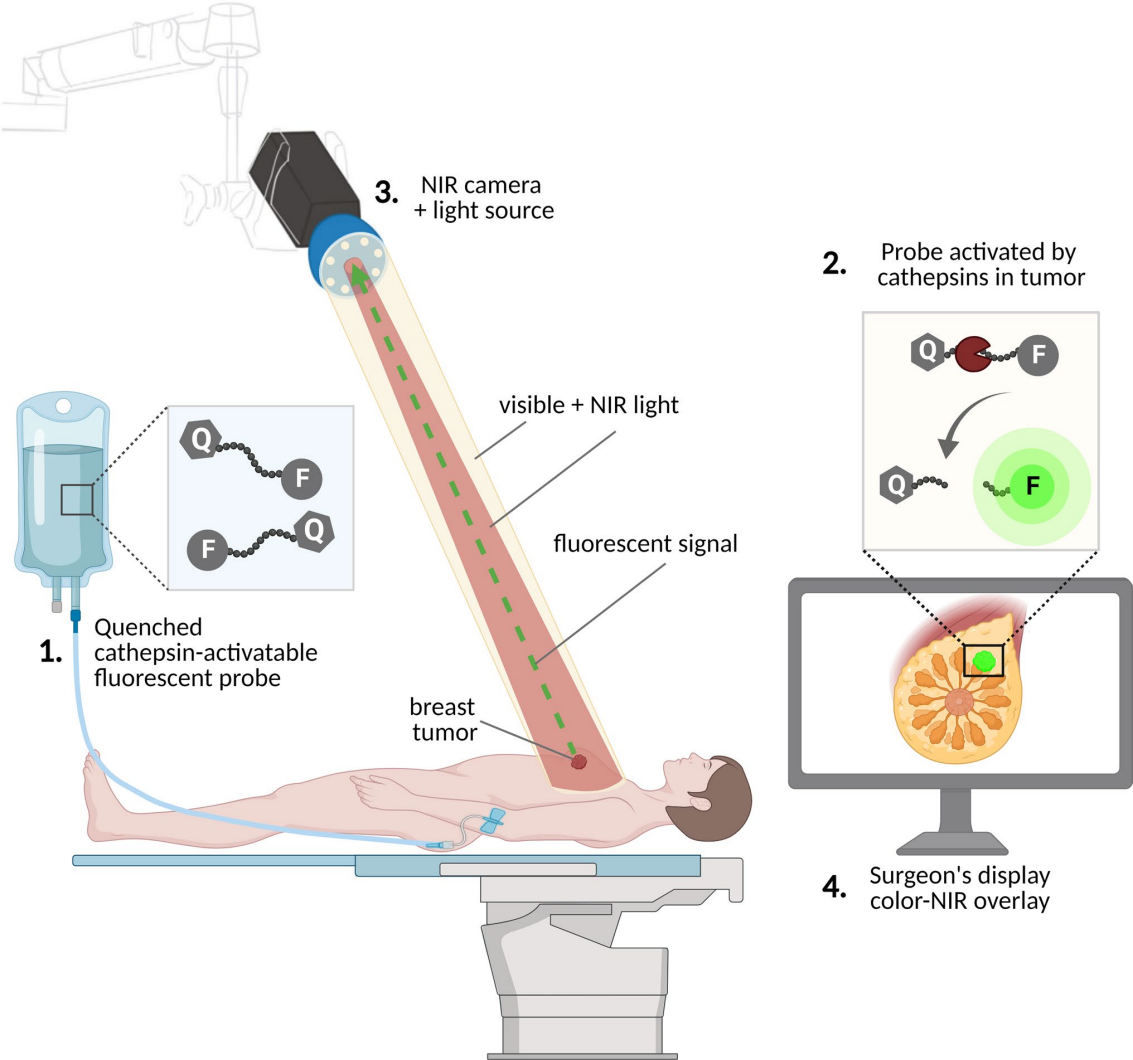
Cathepsin-targeted fluorescence-guided surgery. **1** A quenched, cathepsin-activatable fluorescent probe is administered intravenously prior to surgery or topically during surgery. **2** The probe is activated by cysteine cathepsins overexpressed by tumor and/or stromal cells. **3** The fluorescence signal generated by the activated probe is detected using a NIR sensitive camera system and **4** displayed on a screen in the operating theater. Q, quencher; F, fluorophore; pacman shape, cysteine cathepsin activating the probe; green dotted arrow, fluorescent signal generated by the activated probe in the tumor after illumination with NIR light from the camera system. Abbreviations: NIR, near infrared.

Over recent decades, it has been shown that various members of the cysteine cathepsin family have a more than tenfold overexpression and a more than 50-fold increase in enzymatic activity in breast cancer compared with healthy breast tissue [56–58]. Until recently, the expression of cysteine cathepsins in breast cancer has been assessed mainly for its prognostic value. However, because cysteine cathepsins are upregulated in many breast cancer subtypes, they are also extensively researched as molecular targets [54, 59]. The use of cathepsin-targeted NIR FI during breast cancer surgery could help to identify residual tumor on the surgical cavity surfaces and guide additional excision, thereby minimizing tumor-positive margins, the need for re-resection, and local recurrence (Fig. 1).

In this review, we first discuss the expression of the individual cysteine cathepsins in breast cancer to substantiate their potential use as a target for fluorescence-guided breast cancer surgery. The scope of this review is restricted to the most extensively investigated cysteine cathepsins B, C, K, L, O, S, and V. We summarize their specific role in cancer progression, cellular sources, and correlation to clinicopathological characteristics such as molecular subtype (Table 1). In addition, we will provide an overview of the cathepsin-targeted fluorescent probes that have been investigated for fluorescence-guided breast cancer surgery both preclinically and in patients.

**Table 1:** Cysteine cathepsins in breast cancer

Cathepsin	Main cellular source	Cellular localization	Specific role in metastasis to	Breast cancer subtype	Expression in increasing grade and stage	References
Cathepsin B	Tumor cells Macrophages Fibroblasts Endothelial cells Myoepithelial cells	Lysosomal Extracellular	Lung Bone	IBC HER2 + ER + PR +	Increases	[56, 58, 60–83]
Cathepsin C	Tumor cells (that metastasized to the lung) Leukocytes Fibroblasts	Lysosomal	Lung	ER + PR + HER2 + TNBC	Unknown	[84–87]
Cathepsin K	Tumor cells (that metastasized to the bone) Myofibroblasts	Lysosomal Extracellular	Bone	ER + PR + HER2 + TNBC	Increases	[38, 91–99]
Cathepsin L	Tumor cells Macrophages Myofibroblasts	Lysosomal Extracellular	Lung	ER + PR + HER2 + TNBC	Increases	[56–58, 61, 66, 67, 95, 100–106, 108]
Cathepsin O	Tumor cells	Lysosomal	Unknown	ER +	Unknown	[109–112]
Cathepsin S	Tumor cells Macrophages	Lysosomal Extracellular	Brain	TNBC ER + PR +	Unknown	[30, 33, 69, 95, 105, 113–117]
Cathepsin V	Tumor cells Stromal fibroblasts	Lysosomal	Unknown	ER + HER2 +	Increases	[33, 118–121]

Shown are the cellular sources, cellular localization, specific roles, associated breast cancer subtypes, and the influence of grade and stage on expression for the different cysteine cathepsins discussed in this review

Abbreviations**: **IBC, inflammatory breast cancer; HER2, human epidermal growth factor receptor 2, ER, estrogen receptor; PR, progesterone receptor; TNBC, triple negative breast cancer

## Cysteine Cathepsin Expression in Breast Cancer

### Cathepsin B

Cathepsin B is one of the more ubiquitously expressed members of the cysteine cathepsin family that has been a main focus of research in breast cancer. It has both carboxypeptidase and endopeptidase activity and its substrates include ECM components such as laminin, fibronectin, collagen types I and IV, and proteoglycans [60]. Lah et al. were the first to report significantly higher cathepsin B levels in breast cancer tumors compared with matched normal breast tissue samples [58]. This was confirmed in additional studies, where reports detailed its increased activity across different histological and molecular subtypes of breast cancer [56, 58, 61–64]. Upregulation of this protease is a prognostic factor for (disease free) survival of breast cancer patients, and its enzymatic activity and level of expression have been shown to increase with advancing tumor grade [57, 65–67].

The cellular sources of cathepsin B in breast cancer are predominantly tumor cells and macrophages and, to a lesser extent, stromal components such as myoepithelial cells, fibroblasts, myofibroblasts, and endothelial cells of the neo-vasculature [61, 62]. Both *in vitro* and *in vivo* studies have demonstrated that the cellular origin of cathepsin B overexpression can change during tumor progression [68]. In the primary tumor, cathepsin B derived from tumor cells rather than macrophages promotes cancer progression, while at the metastatic site stromal-derived cathepsin B seems to be the main driver of tumor progression [68–70]. It is well established that expression is not confined to the lysosomal compartment, as translocation of cathepsin B to the cell membrane or secretion into the extracellular space during neoplastic transformation have been reported [71–74]. This redistribution is associated with malignant progression that could be related to exposure of the enzyme to a distinct set of substrates. Additionally, translocation to the cell membrane makes the protease easily accessible to cathepsin B-targeted probes.

The important contribution of cathepsin B to breast cancer progression becomes clear in cell line and murine model studies. Knockdown or selective inhibition of cathepsin B suppresses cancer invasion and metastasis to the lung and bone, whereas an increased cathepsin B expression and activity promotes metastatic spread [75–79].

Several underlying molecular mechanisms of cathepsin B overexpression and its contribution to cancer progression have been elucidated, some of which seem to be breast cancer subtype-specific. In human epidermal growth factor receptor 2 (HER2)-positive breast cancer, for instance, overexpression is the result of an increased transcription of the cathepsin B gene due to an HER2-activated kinase signaling network [80]. In hormonal receptor-positive breast cancer cells, interleukin 6, known for its stimulation of aromatase expression, has been shown to induce cathepsin B expression [81]. *In vitro* studies revealed a functional role of upregulated cathepsin B in pericellular proteolysis of the ECM and basement membrane components, driving tumor invasion [71, 82]. Interestingly, inflammatory breast cancer cells have been identified as particularly benefitting from cathepsin B abundance [56, 58, 61, 62]. Both inflammatory breast cancer cell lines and patient samples show elevated levels of cathepsin B, with the latter displaying a positive correlation with the number of lymph node metastases [83].

In conclusion, cathepsin B is upregulated in various breast cancer subtypes and has a broad spectrum of cellular sources at the primary and metastatic site. It plays a prominent role in many tumor-promoting processes and seems to be specifically implicated in breast cancer metastasis to the lung and bone. Jointly, the data suggest that the upregulation and partly extracellular localization of cathepsin B may be a prime target for imaging purposes.

### Cathepsin C

Cathepsin C is a ubiquitously expressed lysosomal aminopeptidase required for the activation of pro-inflammatory neutrophil serine proteases such as elastase and proteinase 3, signifying its role as a mediator of inflammation [84]. Cathepsin C is necessary for normal mammary gland development [85]. During mammary carcinogenesis, cathepsin C expression and enzymatic activity is elevated, mainly in stromal cells like leukocytes and fibroblasts, but in tumor cells as well [86].

A wealth of information on the specific role of cathepsin C in breast cancer was derived from a series of experiments by Xiao et al., ascribing this enzyme a key role in lung metastasis [87]. In human cell lines derived from primary and metastatic tumors and in tissue samples of different molecular breast cancer subtypes, significantly higher cathepsin C levels in lung metastases than in primary breast tumors were observed. Additionally, human-transgenic mouse orthotopic breast cancer models revealed a higher lung metastasis burden in cathepsin C overexpressing mice and a significantly reduced lung metastasis capacity in the respective knockdown counterparts, indicating a potential therapeutic role for cathepsin C inhibition [87].

The functional role on a molecular level of cathepsin C is related, at least *in vitro*, to induction of signaling pathways that result in neutrophil recruitment and the formation of neutrophil extracellular traps (NETs)—web-like structures composed of granule proteins and decondensed chromatin that promote tumor progression and metastasis [87]. This is in line with the frequently observed exploitation of NETs by cancer cells, promoting their dissemination [88, 89]. The clinical relevance of these findings was highlighted by elevated neutrophil infiltration and NET formation in human lung metastasis as compared with the primary breast tumor and a positive correlation of these factors with cathepsin C expression [87]. Interestingly, the authors reported cathepsin C enzymatic activity and expression and the correlating NET formation to be higher in patients with triple-negative breast cancer (TNBC) compared to hormonal receptor-positive breast cancer.

To summarize, in breast cancer, cathepsin C holds a specialized role during the early stages of pulmonary colonization. By stimulating a signaling cascade leading to the activation and exploitation of neutrophils, this enzyme promotes cancer cell proliferation in the lung. The mainly lysosomal location of cathepsin C could hinder its accessibility to probes, making it less ideal as an imaging target [90]. On the other hand, increased cathepsin C activity and expression in tumor (and associated) cells of all breast cancer subtypes does make this protease a promising target for imaging of lung metastases.

### Cathepsin K

Increasing evidence provides insight into how breast cancer cells take advantage of yet another member of the cathepsin family, cathepsin K. Cathepsin K is an endopeptidase that functions in the lysosomal and extracellular environment [91]. As a potent collagenase, this enzyme has a very specialized function during bone remodeling; thus, under physiological conditions, its expression and secretion are mainly limited to osteoclasts [38, 92]. However, an early study demonstrated that this protease is also expressed in primary breast cancer cells and bone metastases [93]. Subsequent studies confirmed its expression and increased enzymatic activity in bone-residing breast cancer cells and primary tumor cells, while demonstrating its absence in non-cancerous breast tissue and soft tissue metastases [91, 93–96]. Importantly, cathepsin K expression is consistently higher within bone-residing breast cancer cells than in the primary tumor, suggesting that this enzyme has a central role in creating the necessary microenvironmental conditions for breast cancer cells to metastasize to bone.

One of the molecular mechanisms through which cathepsin K contributes to breast cancer progression is by activation of ECM degrading matrix metalloproteinases, which enhances the invasiveness and metastatic capacity of breast cancer cells [97]. Additionally, secreted cathepsin K itself can degrade ECM components [98].

As for associations with other clinicopathological characteristics, assessment of both hormonal receptor-positive and -negative cell lines and patient samples at different stages imply a stage rather than receptor-dependent expression [91, 93, 95, 99].

To conclude, cathepsin K plays a major role in the metastatic spread of breast cancer cells to the bone. Its overexpression and increased enzymatic activity in bone-residing breast cancer cells and its extracellular localization indicates the potential associated with this protease for the imaging of bone metastases.

### Cathepsin L

A considerable number of experiments have been performed to investigate the expression and role of cathepsin L in breast cancer. Cathepsin L is a lysosomal and extracellular endopeptidase whose expression is upregulated in breast cancer cells and TAMs, and which increases with advancing tumor grade [58, 61, 67]. Its overexpression and increased enzymatic activity have been observed across various breast cancer subtypes and is a prognostic factor for (disease free) survival of breast cancer patients [56, 57, 61, 66, 67, 95, 100–102]. Cathepsin L has been shown to play a major role in the process of lung metastasis [101, 103, 104]. *In vitro* ribonucleic acid (RNA) interference and pharmacological inhibition studies demonstrated that breast cancer cells use cathepsin L to enhance their proliferative, invasive, and migratory capacity by degrading ECM components[101–106]. This effect was also observed *in vivo*, as mice injected with cathepsin L knockdown breast cancer cells or treated with a selective cathepsin L inhibitor exhibited significantly smaller tumors compared to the controls with functional cathepsin L [101, 103, 105].

One of the mechanisms by which cathepsin L is upregulated is the loss of stress-induced shutdown of selective messenger RNA (mRNA) translation [103]. Under physiological conditions, cellular stress conditions, such as hypoxia, induce general shutdown of protein biosynthesis [107]. Due to tumor-associated resistance to stress conditions, breast cancer cells can maintain high levels of cathepsin L. This aberrant cathepsin L activity in turn results in upregulation of the mammalian target of rapamycin (mTOR), a key regulator of tumor cell proliferation, tumor growth, survival, and angiogenesis [102]. In addition, it has been shown that the p53 gene is activated by tumor-associated stress conditions, resulting in the downregulation of cystatins and, as a consequence, increased cathepsin L activity [108].

In conclusion, across a variety of breast cancer subtypes significant overexpression and amplified enzymatic activity of cathepsin L is observed, which increases with advancing grade and stage and has an important role during lung metastasis. It has also been established that breast cancer cells can maintain high cathepsin L levels, prioritizing its expression during stress conditions. Both cathepsin L upregulation in breast cancer cells of different molecular subtypes and its partly extracellular localization make cathepsin L a suitable target for intraoperative fluorescence imaging of breast cancer.

### Cathepsin O

Currently, only a limited number of studies have been conducted to examine the expression and role of cathepsin O in breast cancer. Nevertheless, these few studies show that cathepsin O is highly expressed by breast cancer cells [109]. The overexpression is associated with estrogen receptor (ER) status and a decreased response tamoxifen. Genome-wide association studies found overexpression of cathepsin O is caused by variants of small nucleotide polymorphisms (SNP) near the CSTO gene [109–112]. Upregulation of cathepsin O has been shown to facilitate downregulation of the breast cancer 1 gene (BRCA1), by activating protein degradation pathways and through modulation of transcription regulators. Since BRCA1 is important for deoxyribonucleic acid (DNA) double-strand break repair, the upregulation of cathepsin O contributes to the neoplastic transformation of breast cancer cells [109–112].

Its expression in ER-positive breast cancer cells suggests cathepsin O could be a suitable target for tumor imaging. However, cathepsin O expression and activity in tumor cells versus normal breast tissue, its cellular sources and localization should first be more extensively investigated for all breast cancer subtypes.

### Cathepsin S

Cathepsin S is a lysosomal and extracellular endopeptidase [30, 33]. Its overexpression and increased enzymatic activity have been reported in hormone responsive cell lines, yet it is expressed to a much higher extent in the more aggressive TNBC subtype [95, 113–115]. Tumor cells and tumor-stromal macrophages have been identified as the main cellular sources, whereas increased stromal expression is associated with higher tumor grade [115, 116].

Evidence for the contribution of this protease to tumor progression is reflected by the profound impact of knockdowns and pharmacological inhibition in TNBC cell lines as well as in animal models [105, 114, 116]. These studies show that interference with cathepsin S activity using small-interfering RNA or targeted inhibition prevents invasion and metastasis of breast cancer cells. This is consistent with impeded invasive and migratory capacity in highly metastatic cell lines upon cathepsin S inhibition [105].

Cathepsin S has been shown to have a prominent role in orchestrating breast to brain metastasis [114, 116]. During the early stages of brain metastasis, the main cellular source for cathepsin S are tumor cells, with limited stromal cell contribution. This pattern shifts during late stages, where stromal cells become the main cellular source and tumor-derived cathepsin S expression decreases. However, the experimental downregulation of both sources is required to limit metastasis. The underlying molecular mechanism of cathepsin S-driven brain metastasis is the proteolysis of the junctional adhesion molecule B, expressed on the blood–brain barrier. Breast tumor cells equipped with cathepsin S are capable of migrating across this strict and highly selective barrier by enabling the cleavage of these restricting junction proteins [116].

Another mechanism by which cathepsin S contributes to breast cancer progression is the proteolytic degradation of the aforementioned BRCA1, resulting in suppressed DNA double-strand break repair activity [117]. Contrary to expectation, this does not make breast cancer cells more susceptible to chemotherapy. In fact, cathepsin S overexpression in the tumor seems to inhibit the effects of chemotherapy on breast cancer cells, possibly by enabling TAMs to provide survival signals [69].

Taken together, these studies indicate the relevance of cathepsin S in the progression of breast cancer cells, especially TNBC, and show its contribution to brain metastasis by facilitating the crossing of the blood–brain barrier. Increased cathepsin S expression and activity in breast cancer and its part extracellular localization would make this enzyme a perfect target for the tumor imaging of TNBC and its brain metastases.

### Cathepsin V

Cathepsin V is a mainly lysosomal endopeptidase that shares structural similarities with cathepsin L; however, it exerts distinct functions and has a confined tissue distribution [33]. Under physiological conditions, this protease is primarily expressed within the thymus, testis, and corneal epithelium [118]. Data on its involvement in breast cancer is scarce; however, an early study has demonstrated elevated expression in breast cancer cell lines and tissues compared with non-cancerous breast tissue [118].

More recently, cathepsin V expression has been associated with advancing tumor grade, distant metastasis, and breast cancer recurrence [119, 120]. Expression of cathepsin V has been demonstrated in different molecular subtypes of breast cancer and seems to increase during the transition of ductal carcinoma in situ (DCIS) to invasive breast carcinoma, suggesting this protease contributes to the tumor’s invasiveness [121]. Because of its role in tissue invasion, cathepsin V has been included in the genetic signature list for the oncotype DX, an array that comprises the expression of 21 genes and is used to quantify the likelihood of distant recurrence in the ER-positive sub-population [120].

On the molecular level, cathepsin V promotes the degradation of GATA binding protein 3 (GATA3) in ER-positive breast cancer cells [119]. GATA3 is a crucial transcription factor for the normal development of mammary glands and its depletion has been strongly associated with breast cancer progression due to loss of normal cellular differentiation, adhesion, and proliferation [122, 123]. This is in line with the finding that high GATA3 levels are associated with better outcomes in ER-positive patients [124].

In summary, cathepsin V seems to play an important role in the progression from DCIS towards invasive breast cancer. The available data indicates that elevated cathepsin V levels promote the progression of ER-positive cancer cells by degradation of GATA3. Cathepsin V upregulation in breast cancer cells compared to normal breast tissue indicates that this protease could be useful for targeted FI. A possible disadvantage is its mainly lysosomal localization, which could make it less accessible to an imaging probe.

## Cathepsin-Targeted Probes for Fluorescence-Guided Breast Cancer Surgery

In recent decades, numerous cathepsin-targeted FI agents have been developed. Initially, cathepsin-targeted probes were used to visualize cathepsin activity *in vitro* to study their role in cellular (patho)physiology. However, since the introduction of clinical NIR FI systems, cathepsin-targeted probes are under extensive investigation for their possible use in intraoperative tumor visualization. Because of the increased expression and proteolytic activity of cysteine cathepsins in breast tumors of virtually all molecular subtypes, these proteases show great potential as targets for fluorescence-guided breast cancer surgery in a large patient population. Moreover, since cysteine cathepsins derive from both tumor cells and tumor-stromal cells, such as TAMs, cathepsin-targeted probes will potentially result in a more homogeneous tumor signal compared to probes targeting tumor cell-specific proteins, such as epidermal growth factor receptor (EGFR), that often display high intratumor and interpatient heterogeneity [125, 126].

**Fig. 2:**
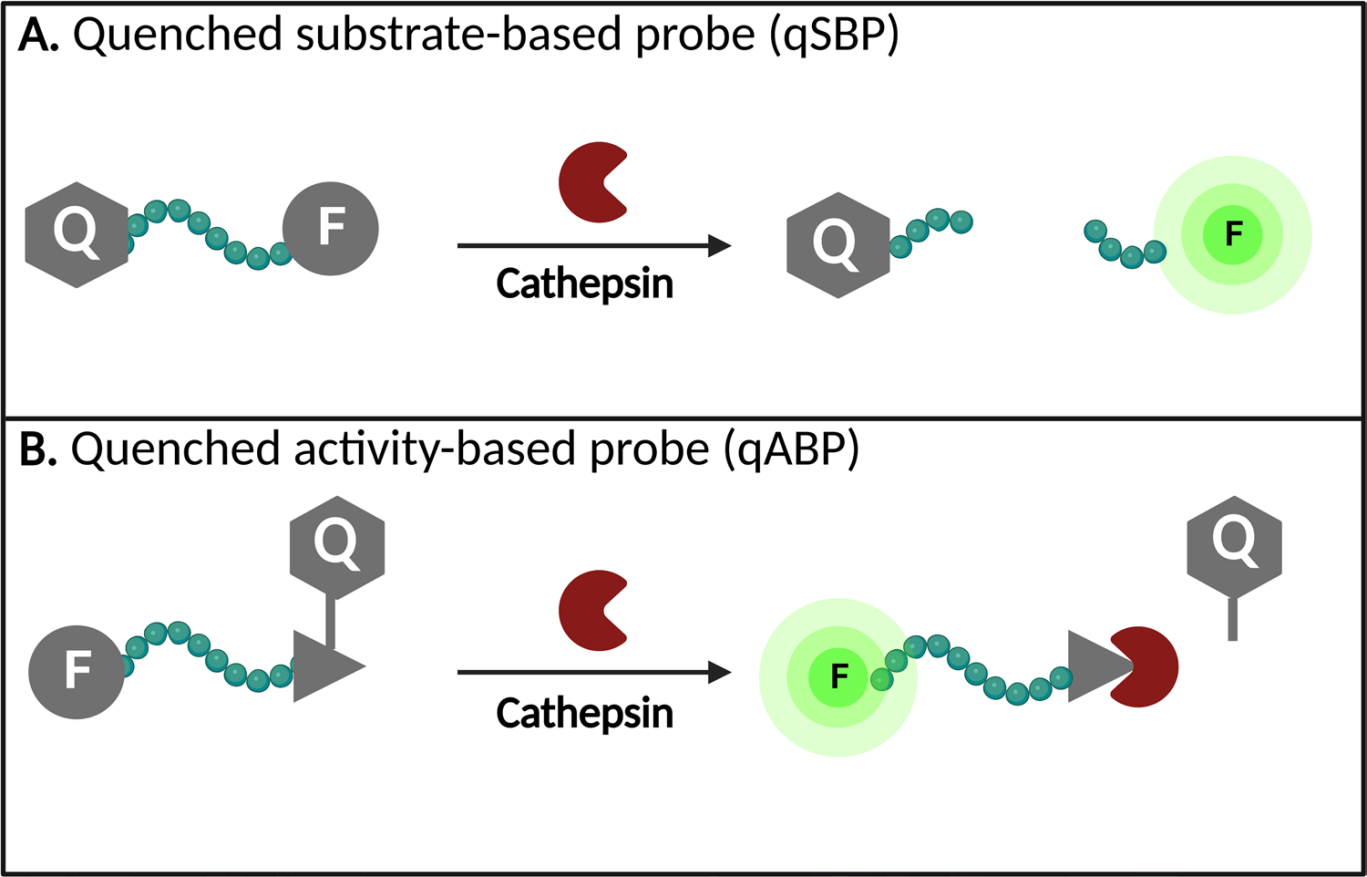
Different types of quenched cathepsin-activatable fluorescent probes. **A** Quenched substrate-based probe. The probe is activated by enzymatic cleavage of the peptide linker by a target cathepsin. Upon cleavage, two fragments—one containing the quencher and the other the now unquenched fluorophore—are released. **B** Quenched activity-based probe. The probe covalently binds in the active site of the target cathepsin forming a permanent bond. Upon binding the active site, the quencher is released and the probe is activated. Abbreviations: qSPB, quenched substrate-based probe; qABP, quenched activity-based probe; Q, quencher; F, fluorophore.

Most cysteine cathepsin-targeted contrast agents are so-called turn-ON (or *quenched)* probes that only fluoresce after activation. They can either be activated by one specific cathepsin or be pan-reactive, targeting multiple cysteine cathepsins at once. The advantage of activatable agents over traditional fluorophores is the substantial reduction in off-target (or *background*) fluorescence and an increased speed of detection since no time is required to clear from non-target tissues [127]. The activatable cathepsin-targeted fluorescence agents can be subdivided into substrate-based probes (SBP) and activity-based probes (ABPs). SBPs require enzymatic cleavage of the probe’s substrate to become activated, while ABPs form covalent bonds at the catalytic site of cathepsins and therefore do not diffuse from the target enzyme after activation, resulting in prolonged signal retention at the target location (Fig. 2) [59].

Completed and onging clinical trials have shown that the process from NIR fluorescence probe development to first-in-human trials and subsequent clinical translation is expensive and time-consuming, stressing the imporance of selecting the probes with the most potential in preclinical research (NCT03659448) [128]. The following sections will therefore review both cathepsin-targeted probes that have only been investigated in preclinical research as well as cathepsin-targeted contrast agents that have already been used for fluorescence-guided breast cancer surgery in patients. The details of these probes are summarized in Table 2.

**Table 2:** Cysteine cathepsin-targeted fluorescence probes in breast cancer

Probe name	Probe type	Target	Fluorophore	Phase	References
BMV083	qABP	Cathepsin S	Cy5	Preclinical	[129]
BMV109	qABP	Cathepsin B, L, and S	Cy5	Preclinical	[130, 131]
VGT-309	qABP	Cathepsin B, L, and S	ICG	Preclinical	[132]
BMV157	qABP	Cathepsin S	Cy5	Preclinical	[133]
6QC-NIR	qSBP	Cathepsin B, L, and S	DyLight780-B1	Preclinical	[134]
6QC-ICG	qSBP	Cathepsin B, L, and S	ICG	Preclinical	[135, 136]
DEATH-CAT-FNIR	AND-Gate	Cathepsin L (and caspase 3)	Heptamethine cyanine	Preclinical	[136]
YBN14	Photosensitized qABP	Cathepsin B, L, and S	Bacteriochlorin	Preclinical	[137]
8h6	DARPin	Cathepsin B	Cy5.5	Preclinical	[138]
*Not specified*	qABP	Cathepsin S	Cy5.5	Preclinical	[139]
MP-cL3	ABP	Cathepsin L	Cy5	Preclinical	[140]
LUM015	qSBP	Cathepsin B, K, L and S	Cy5	Preclinical/clinical	[23, 141–143] NCT03321929 NCT03686215

Shown are the cathepsin-targeted fluorescent probes for intraoperative breast cancer imaging that have been investigated preclinically and in human patients

Abbreviations: *ABP*, activity-based probe; *qABP*, quenched activity-based probe; *qSBP*, quenched substrate-based probe; *AND-Gate*, probe that requires activation by two different targets; *DARPin*, designed ankyrin repeat protein; *Cy5*, cyanine 5; *ICG*, indocyanine green; *Cy5.5*, cyanine 5.5.

### Preclinical

Various quenched substrate-based (qSBP) and activity-based probes (qABPs) have been investigated in breast cancer cell lines and mouse models. The cathepsin S-directed qABP BMV083, developed by Verdoes et al., allowed for *in vivo* breast cancer visualization in a syngeneic orthotopic mouse breast cancer model [129]. Withana et al. showed that an altered version of this probe, BMV109, targeting both cathepsin B, L, and S, had enhanced imaging properites *in vivo.* In addition, it enabled rapid imaging of enzyme activity in fresh frozen human breast cancer tissue sections [130, 131]. Suurs et al. developed the cathepsin B-, L-, and, S-targeted probe VGT-309, which contains the same cathepsin recognition sequence as BMV109 but a different fluorophore and quencher. In a syngeneic orthotopic breast cancer mouse model, tumors were well delineated *in vivo* by the fluorescent signal and VGT-309 could be used for fluorescence-guided resection [132]. Bender et al. designed a potent cathepsin S-targeted qABP, BMV157, that could demarcate breast cancer margins with substantial contrast to surrounding healthy tissue *in vivo* in a syngeneic orthotopic mouse model. However, due to the selectivity of BMV157 for cathepsin S, its fluorescence signal in breast tumors was weaker than that of BMV109 [133].

Further improvement of BMV109 by Ofori et al. resulted in the quenched substrate-based probe 6QC-NIR, designed to exploit the latent lysosomotropic effect [134]. This causes accumulation of the fluorescent fragments of the probe in lysosomes following proteolytic cleavage by cathepsin B, L, or S, preventing diffusion from the target (tumor) location. In a syngeneic orthotopic mouse model of breast cancer, the use of 6QC-NIR provided evidence of its ability to visualize tumors and guide resection using real-time intraoperative FIwith the FDA-approved da Vinci Surgical System and its integrated NIR fluorescence camera system (Firefly mode; Intuitive Surgical, Sunnyville California, USA). Moreover, the use of cathepsin-targeted FI with 6QC-NIR allowed detection of additional breast cancer lesions that were invisible under white light [134]. Yim et al. further optimized the 6QC-NIR probe by replacing the fluorophore Dylight 780-B1 with the FDA-approved fluorophore indocyanine green (ICG). Compared to 6QC-NIR, 6QC-ICG had an enhanced fluorescence signal and an improved sensitivity during fluorescence-guided breast cancer surgery in a syngeneic orthotopic mouse model using commercially available FI cameras that were optimized to detect ICG [135].

To increase tumor selectivity, Widen et al. synthesized a so-called AND-Gate probe, DEATH-CAT-FNIR, that required activation by cathepsin L and caspase 3, a different type of protease [136]. This AND-Gate design diminishes the off-target signal by requiring both processing events to be present in the same location. During fluorescence-guided breast cancer surgery using the da Vinci Firefly in a syngeneic orthotopic mouse model, the DEATH-CAT-FNIR had comparable fluorescence signal intensity to the 6QC-ICG probe and a much improved signal in comparison to 6QC-NIR. In addition, DEATH-CAT-FNIR demonstrated a lower background signal in healthy organs and facilitated detection of residual breast cancer cells after resection [136].

Ben-Nun et al. developed a cathepsin B, L, and S targeted, photosensitized qABP. This probe, YBN14, was able to visualize breast tumors *in vivo* in a syngeneic subcutaneous mouse model and was succesfully used for photodynamic therapy (PDT), a method for cancer treatment that involves the activation of a photosensitive molecule by a light source to cause selective cytotoxic damage to cancer cells [137]. Kramer et al. constructed DARPin 8h6, a highly selective cathepsin B-directed, fluorescently labeled *designed ankyrin repeat protein* (DARPin), which is a small antibody mimetic. In both a congenic and syngeneic orthotopic breast cancer mouse model, DARPin 8h6 highlighted mammary tumors both in and *ex vivo* with a considerable contrast to healthy tissue [138]. A cathepsin S-directed, lipidated qABP was designed by Hu et al. and has been demonstrated to visualize breast tumors *in vivo* with a high tumor to background ratio [139]. Porreba et al. developed the cathepsin L selective ABP MP-cL3, which has been shown to label breast cancer cells *in vitro* [140]. The PEGylated cathepsin K-, L-, and S-targeted qSBP probe LUM015, designed by Whitley et al., exhibited an increased fluorescence signal *ex vivo* in breast cancer compared with normal muscle tissue in an orthotopic mouse model [141].

### Clinical

To the best of our knowledge, LUM015 is the only cathepsin-targeted contrast agent for fluorescence-guided breast cancer surgery that has been introduced in the clinic.

A first-in-human phase I clinical trial showed that preoperative intravenous LUM015 administration did not raise safety signals and resulted in tumor-specific fluorescence that could be detected upon *ex vivo* imaging of resected breast cancer tissues [141]. A subsequent dose-escalation pilot study demonstrated that LUM015 allows direct identification of residual tumor in the breast cancer patient’s surgical cavity with a high tumor-to-background ratio of the fluorescent signal [23]. In a phase II clinical trial of 45 breast cancer patients who underwent BCS, the LUM015 fluorescence signal facilitated detection of 84% of all tumor-positive cavity surfaces in the eight patients with tumor-positive margins. In addition, two patients with tumor-positive margins after standard of care surgery were spared second surgeries because additional tissue was excised at sites of high LUM015 signal [142]. In an additional phase II clinical trial of 55 breast cancer patients undergoing BCS, the tumor-to-background ratio of the fluorescence signal ranged between 3.8 and 5.7 [143]. An ongoing multicenter phase II clinical trial (NCT03321929) and a phase III randomized controlled trial (NCT03686215) will further investigate the feasibility and added clinical value of LUM015 for the intraoperative detection of residual tumor during breast cancer surgery.

The previously described cathepsin-targeted probe VGT-309 is currently under investigation in a phase II clinical trial in 40 patients undergoing surgery for primary lung cancer or lung metastases (NCT05400226). The preliminary results in two patients have already been published and illustrate the successful clinical translation of VGT-309 and its potential to improve surgical management of patients undergoing cancer resection [144]. However, VGT0309 has not yet been tested in breast cancer patients.

## Future Perspectives: Topical Application of Cathepsin-Targeted Imaging Probes

To date, cathepsin-targeted activatable imaging probes for fluorescence-guided breast cancer surgery have been administered intravenously, necessarily days or hours prior to surgery. Alternative to systemic delivery, cathepsin-targeted turn-ON probes could be topically applied onto the breast cancer patient’s surgical cavity to rapidly differentiate between tumor and healthy tissue, as has been demonstrated in a recent preclinical study [145]. The main advantages of topical application compared with intravenous administration are (1) faster probe activation (minutes instead of days/hours), hence more compatible with normal surgical workflow, (2) a better identification of tumor cells at the resection margin that have not necessarily generated a vascular system yet, (3) a decreased chance of side effects due to a diminished systemic load, (4) a potentially more cost-effective probe development process due to the possibility of using a micro-dose, and (5) it offers a more patient-friendly approach, as patients do not have to visit the hospital prior to surgery for IV injection [146].

## Conclusions

In this review**,** we have discussed the expression and role of cysteine cathepsins in breast cancer and their applicability to fluorescence-guided breast cancer surgery. The cysteine cathepsins B, C, K, L, O, S and V are all highly overexpressed by and have an increased proteolytic activity in virtually all breast cancer subtypes and play major roles in tumor progression. Most of these proteases have been shown to be suitable targets for fluorescence-guided breast cancer surgery, a technique that can identify tumors, facilitate residual tumor identification and guide additional resection. The first and only cathepsin-targeted probe introduced in the clinic thus far shows promising results, possibly preventing the need for re-excision and—potentially—tumor recurrence. Various other cathepsin-targeted probes demonstrate great potential for intraoperative breast cancer imaging in preclinical studies. Due to their particular design, some of these probes offer certain advantages, such as retention of the fluorescence signal in the target cells or a lower background signal, probably making them the most preferable agents for fluorescence-guided breast cancer surgery. Clinical translation of these promising cathepsin-targeted fluorescent probes in the near future could greatly improve treatment outcomes for breast cancer patients, especially if used in a topical application protocol that is more patient friendly and easily integrated into the surgical workflow.
